# Poly(propylene fumarate) Composite Scaffolds for Bone Tissue Engineering: Innovation in Fabrication Techniques and Artificial Intelligence Integration

**DOI:** 10.3390/polym17091212

**Published:** 2025-04-28

**Authors:** Madalina I. Necolau, Mariana Ionita, Andreea M. Pandele

**Affiliations:** 1 Advanced Polymer Materials Group, National University of Science and Technology Politehnica Bucharest, Gh. Polizu Street, 011062 Bucharest, Romania; madalina.necolau@upb.ro (M.I.N.); mariana.ionita@upb.ro (M.I.); 2 Department of Analytical Chemistry and Environmental Engineering, National University of Science and Technology Politehnica Bucharest, Gh. Polizu Street, 011062 Bucharest, Romania

**Keywords:** tissue engineering, artificial intelligence, poly(propylene fumarate), composite scaffolds

## Abstract

Over the past three decades, the biodegradable polymer known as poly(propylene fumarate) (PPF) has been the subject of numerous research due to its unique properties. Its biocompatibility and controllable mechanical properties have encouraged numerous scientists to manufacture and produce a wide range of PPF-based materials for biomedical purposes. Additionally, the ability to tailor the degradation rate of the scaffold material to match the rate of new bone tissue formation is particularly relevant in bone tissue engineering, where synchronized degradation and tissue regeneration are critical for effective healing. This review thoroughly summarizes the advancements in different approaches for PPF and PPF-based composite scaffold preparation for bone tissue engineering. Additionally, the challenges faced by each approach, such as biocompatibility, degradation, mechanical features, and crosslinking, were emphasized, and the noteworthy benefits of the most pertinent synthesis strategies were highlighted. Furthermore, the synergistic outcome between tissue engineering and artificial intelligence (AI) was addressed, along with the advantages brought by the implication of machine learning (ML) as well as the revolutionary impact on regenerative medicines. Future advances in bone tissue engineering could be facilitated by the enormous potential for individualized and successful regenerative treatments that arise from the combination of tissue engineering and artificial intelligence. By assessing a patient’s reaction to a certain drug and choosing the best course of action depending on the patient’s genetic and clinical characteristics, AI can also assist in the treatment of illnesses. AI is also used in drug research and discovery, target identification, clinical trial design, and predicting the safety and effectiveness of novel medications. Still, there are ethical issues including data protection and the requirement for reliable data management systems. AI adoption in the healthcare sector is expensive, involving staff and facility investments as well as training healthcare professionals on its application.

## 1. Introduction

Bone tissue represents the second-most frequently transplanted tissue globally due to its high rates of susceptibility to trauma and fractures. Apart from that, the rising incidences of orthopedic-related disorders such as osteoporosis and bone tumors have imposed the necessity to develop efficient alternatives to conventional treatments [1,2]. Tissue engineering (TE) is an interdisciplinary domain that aims to create viable supports that can generate, modify, or even replace diseased or damaged tissue and has recently emerged as a promising solution to overcome all the limitations associated with conventional therapies designed to repair bone [3,4,5].

Currently, bone tissue damage can be treated by employing two protocols based on autologous and allogeneic grafts, imposing the need for a donor [6]. However, regardless of the graft type employed, there are some limitations, such as high risk for the patient to develop an immune response, need for additional procedures, donor site morbidity, and limited number of donors. When designing a bone tissue substitute, it is essential to take into consideration the hierarchical and complex morphology that supports its diverse mechanical, biological, and chemical functions [7]. Considering this, the main goal of tissue engineering is to design functional substitutes for specific tissues, scaffolds, in which living cells can be seeded to assist tissular remodeling [3] either in vivo or with the aid of implanted synthetic or semi-synthetic matrices. Moreover, biomechanical properties, as well as three-dimensional (3D) architecture and extracellular matrix (ECM), must all be taken into account [4,5]. Scaffolds can be synthesized using various biomaterials to achieve specific properties such as high porosity, biocompatibility, and permeability that will further be able to mimic the extracellular matrix of specific tissues [8]. Used either as cell-free microenvironments or as carriers for cells or bioactive molecules, scaffolds represent the ideal structures that can support tissue reconstruction [9].

Numerous resources have been used in an effort to address medical issues throughout history. These substances, which are referred to as “biomaterials”, are composed of numerous different chemicals and are the main constituents used to develop a matrices that can provide a proper microenvironment for cell differentiation and proliferation [10,11].

Scaffold materials have not been widely used in clinical settings, despite the fact that tissue-engineered constructions are developing more quickly. This is because more stringent regulatory permissions are needed to introduce more sophisticated biomaterials into clinical practice [12]. The laws governing the safety of biomaterials can vary greatly from one nation to another and can be highly perplexing when it comes to research involving both human and animal participants. The procedure of getting approval and a license to sell biomaterial goods can also be quite difficult. The primary worldwide organization in charge of guaranteeing product safety and quality is the worldwide Standards Organization (ISO). ISO 10993 describes the biological evaluation of biomaterials and medical devices. These include guidelines for test selection, requirements for animal welfare, various pertinent in vitro and in vivo biological tests, sterilization standards, clinical trials, evaluation and quantification of biodegradation, and material characterization [13].

Compared to the average of all quarters over the previous three years, the European market had about 23.1% rise in medical device recalls in the first quarter of 2023. The new Medical Device-In Vitro Diagnostic Medical Devices (MD-IVD) laws and stakeholders in the European market face a big challenge as a result of this development. After analyzing the situation over the past two decades, noticing regulatory blind spots and a downward trend in device approval, the EU updated its legal foundations. Additionally, Medical Device Regulation (MDR) and In Vitro Diagnostic Regulations (IVDR) were transformed from directives to regulations, making them direct laws in the EU’s member states (MS).

A very important aspect that it is needed to be taken into consideration prior to clinical usage of the biomaterials is sterilization. The goal of sterilization is to lower the danger of infection of the scaffold tissue culture system in vitro or the implanted material in vivo, as well as to eradicate the possibility of disease transfer to people. When choosing a sterilization method for a given biomaterial, it is important to take into account how different methods affect the scaffolds’ biochemical, structural, and physical characteristics, such as their mechanical strength, porosity, and morphology, as well as how residual toxic chemical sterilizing agents affect the biomaterial’s biocompatibility. A recent study by Dai et al. examined various methods used to sterilize biomaterials, including their respective benefits and drawbacks, impacts on biomaterials, efficiency, and mechanisms of action [14].

When it comes to bone tissue engineering, natural polymers such as collagen [15] and gelatin [16] are used due to their natural prevalence within the tissue, mostly in combination with synthetic biopolymers such as poly(lactic acid) (PLA) [17], poly(ε-caprolactone) (PCL) [18], polylactide (PDLA, PLLA) [19,20], and poly(lactide-co-glycolide) (PLGA) [21] that are preferred due to their higher mechanical resistance [22,23,24]. Apart from the mechanical performances, synthetic polymers have numerous advantages over natural materials since their characteristics can be altered to meet certain design specifications, including those for biocompatibility, biodegradability, extremely porous and interconnected structures, high surface area to volume ratios, suitable surface chemistry, and mechanical properties. The high processibility of biodegradable synthetic polymers makes them appealing as scaffold materials for bone tissue engineering [25,26,27,28]. Porosity is a key factor in scaffold design for bone tissue engineering, and it has a signiffincant influences not only over the cell infiltration and vascularization but also on the nutrient diffusion and mechanical strength. Scaffolds with porosity greater than 40% are considered favorable for bone tissue applications while pore sizes exceeding 300 μm are recommended to enhance new bone formation and capillary development [29,30]. Table 1 displays the porosity values that can be achieved by employing some of the most common used synthetic polymers to fabricate scaffolds for bone tissue engineering.

**Figure 1:**
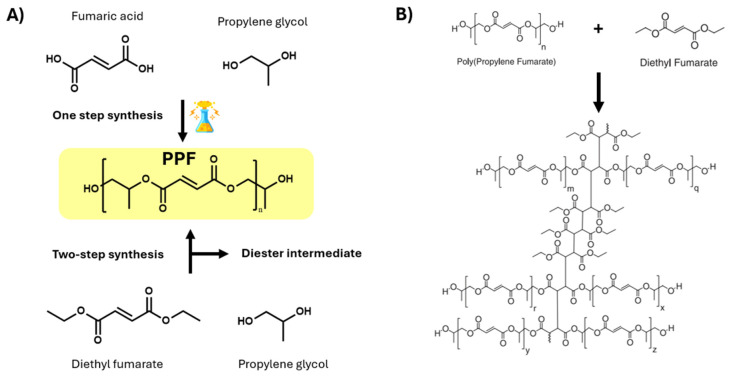
Reaction scheme for the synthesis of PPF (**A**) and chemical crosslinking of PPF with diethyl fumarate (**B**). Reproduced after reference [40].

Poly(propylene fumarate) (PPF) is one of the most attractive polymers in biomedical research, extensively used for the synthesis of scaffolds for bone tissue engineering because of its greater biocompatibility and biodegradability. PPF is a linear unsaturated polyester with double bonds along its backbone that can be synthesized by employing two approaches: one-step methods and multistep methods (Figure 1) [35,36]. The first method comprises the synthesis of PPF starting from fumaryl chloride. This method has as major disadvantage the formation of numerous by-products, which, in the case of polymers for biomedical applications, can considerably increase the toxicity [36,37]. As a consequence of the disadvantage associated with the direct route, the two-step protocol is preferred for the synthesis of PPF, where diethyl fumarate and propylene glycol are reacted together [35]. In the first step bis(hydroxypropyl) fumarate as intermediate product is formed, which will further on undergo transesterification reaction in the second step leading to PPF formation.

In terms of chemical structure, the repeating unit of PPF contains a single free rotating carbon–carbon bond provided by the propylene glycol component and a carbon–carbon double bond that can be further used to obtain 3D networks through crosslinking. There are various methods for the crosslinking of PPF, which implies free radical polymerization using monomers such as methyl methacrylate (MMA) [38], N-vinylpyrrolidinone (NVP) [39], diethyl fumarate (DEF) [40], or with the aid of specific photoinitiators such as bisacylphosphine oxide (BAPO) [41]. BAPO is preferred over other photoinitiators due to its high compatibility with PPF, high reactivity, and efficient radical generation while exerting lower cytotoxicity. Apart from that, its polymerization kinetic can be adjusted by means of light intensity and wavelength, making it ideal for controlled fabrication techniques such as 3D printing [42,43].

Since its introduction by Mikos and Yaszemski, poly(propylene fumarate) (PPF) has been employed pre-clinically for bone tissue engineering, among other synthetic polymers [44]. An important advantage given by PPF for its use in tissue engineering consists in its chemical versatility, which endows the possibility to be processed into a variety of forms with particular pore morphologies, which makes it simple to incorporate various bioactive molecules [44,45]. The use of PPF-based materials has been investigated over time for various biomedical applications such as cardiac tissue engineering, ophthalmology, drug delivery, and neural tissue engineering in addition to their utility in bone tissue engineering research [46].

PPF resorption has been found to occur within a time window relevant to bone remodeling and healing processes, in contrast to other materials that decay either too rapidly or too slowly. The degradation mechanism of PPF relies on the hydrolysis of the ester bonds into nontoxic fumaric acid, poly(acrylic acid-co-fumaric acid), and polypropylene glycol (PG). However, the crosslinking agent used, as well as the crosslinking density and molecular mass, represent important parameters with a strong influence over the degradation process that need to be taken into consideration [47,48]. Nettleton et al. concluded in their study that a polymer with a lower molecular mass is ideal for rapidly healing a bone defect [49]. In the study, two distinct molecular mass resin formulations, 1000 and 1900 Da, respectively, were used to 3D print scaffolds that were further implanted in a rat critical-sized cranial defect. According to the micro-CT analysis, after 4 weeks of implantation, the polymer having the 1000 Da molecular mass degrades faster, but after 12 weeks of implantation, no significant differences have been observed between the two formulations.

The present review comes as a continuation and complements the aspect comprised in the work of Cai et al. centered especially on the recent applications of polypropylene fumarate used as a polymeric matrix for the development of advanced nanocomposite for bone tissue regeneration [43]. One of the objectives in the present work consists in the scaffold design library and properties both for pure PPF scaffolds and the PPF composite scaffolds using various reinforcing fillers by highlighting how scaffold architecture, material formulation, and fabrication strategy critically impact the structural, mechanical, and biological performance of tissue-engineered constructs. Another objective is the use of artificial intelligence aided to the manufacturing strategies. Additionally, the integration of AI with various scaffold development methods facilitates the creation of materials tailored to individual patients’ needs, resulting in feedback and sufficient data for reproducibility that may be improved upon in the future.

## 2. Fabrication of Scaffolds for Tissue Engineering Based on PPF

A crucial aspect that needs to be considered for the successful design of an efficient scaffold consists in selecting the right materials as well as the most suitable manufacturing processes. Porogen leaching [50] and gas foaming [51] are examples of traditional methods through which porous scaffolds for bone tissue engineering can be constructed. However, they provide very little control over essential parameters such as surface area, porosity, and open pores size. These methods are often associated with stochastic porosities that will eventually lead to numerous limitations, such as uneven mass transfer, stress distribution, deterioration, fragmented void regions, and difficulties to achieve repeatability for the synthesis. Considering this, the development of porous scaffolds with complex architectures has registered a tremendous advancement along with the progress of 3D printing methods. Fused deposition modelling (FDM), selective laser sintering (SLS), stereolithography (SLA), electron beam melting (EBM), 3DP technology, and biological 3D printing are currently the primary 3D printing technologies utilized in the creation of bone tissue scaffolds [52]. Table 2 highlights the main techniques used for the design of PPF-based scaffolds along with key parameters.

Stereolithography (SLA) is an additive manufacturing light-based 3D printing technique that involves the polymerization of a photosensitive liquid polymer to produce layer by layer three-dimensional constructs with controlled microstructures [61,62]. It is a method that often yields the highest resolution for porous scaffolds designed for bone tissue engineering [63,64]. This technique gained a lot interest recently along with which significantly increased resolution and reduced the printing time [65,66].

**Figure 2:**
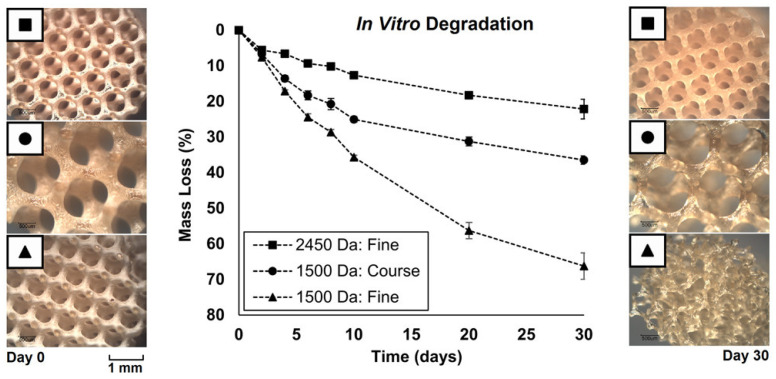
Influence of molecular mass and pore structure on mass loss of PPF-based scaffolds under accelerated degradation and optical micrograph of scaffolds before (**left**) and after (**right**) degradation in 0.1 M NaOH solution for 30 days. Reprinted with permission from [67].

Walker et al. presented in their paper the use of additive manufacturing technique in DMD-based mask projection stereolithography to fabricate scaffolds for tissue engineering from PPF [67]. The authors analyzed the influence of the molecular weight of the polymer over the mechanical performances of the porous scaffolds synthesized through this technique. As can be observed in Figure 2, among the tested groups, 1500-F scaffolds degraded the fastest, while 2450-F scaffolds showed the slowest degradation, retaining most of their mass after 30 days. The optical micrographs reveal that scaffold degrade significantly over time and is strongly influenced by polymer molecular weight and architecture. 1500-F scaffolds exhibited the most noticeable degradation, with substantial strut thinning and loss of structural integrity after 30 days of immersion, while 2450-F scaffolds maintained its structure with minimal visible. The results show that the molecular mass of the polymer not only influences the degradation rate and mechanical performances of the scaffold but also the contraction after polymerization. Thus, high molecular mass (2450 Da) PPF-based scaffolds demonstrate the most notable lower yield stress of about 0.35 MPa and a compressive stiffness of 5 MPa. The study reveals that reproductible scaffold construction is possible. At the same time, by modifying the physical characteristics of the scaffold, the mechanical qualities and degradation kinetics may be tailored as well.

**Figure 3:**
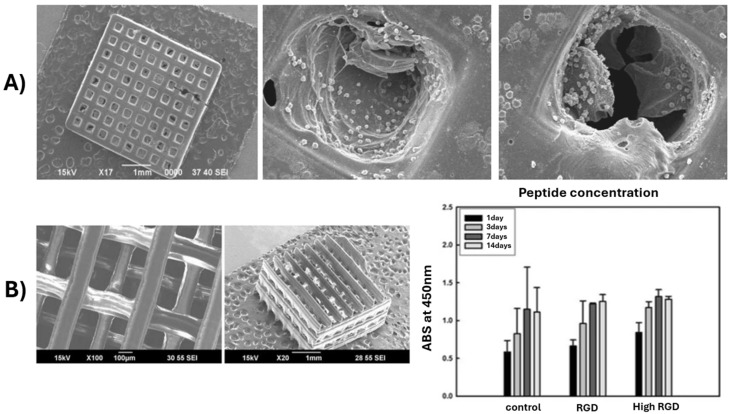
SEM micrographs of PPF/DEF synthesized by using microstereolithography: (**A**) Attachment of the 3T3-L1 mouse fibroblasts cells onto 3D-printed PPF scaffold. Reprinted with permission from Ref. [47]; (**B**) PPF scaffold with a staggered arrangement of line reproduced from Ref. [69].

Lately, stereolithography was replaced by microstereolithography (μSLA), a modern form of stereolithography mainly used for the fabrication of highly detailed structures with micrometric-level resolution. This technique represents a valuable tool to design constructs with extremely fine characteristics (about 2 µm resolution), as mostly required by the biomedical field applications. One of the most important aspects that strongly influences this process and represents a requirement at the same time is the viscosity of the resin, which needs to be reduced. Lee et al. [68] reports the synthesis of a biodegradable PPF resin-based scaffold through a modified synthetic process using micro stereolithography using diethyl fumarate (DEF) to reduce the viscosity of the PPF. The authors concluded that the increase in the concentration of PPF in the liquid resin influences the line width of the layers during printing. However, the designed scaffolds showed good proliferation and adherence of the cells (Figure 3A) [68]. Lee et al. [42] used the same approach to modulate the viscosity of PPF by using DEF, and additionally, they evaluated its influence over the crosslinking of the polymer. It was observed that, when DEF concentration increased over 50 wt. %, the mechanical properties of the final formulation dropped significantly even though the viscosity met the fabrication specifications.

The ideal concentration for PPF to approach good printability and also good mechanical properties was determined as 60–70 wt. % for a polymer with a molecular weight of 2016 g/mol. When the mechanical properties of the scaffolds were correlated with the porosity, it was demonstrated that the compressive modulus increased to 140 MPa as pore sizes decreased to 446 µm [42]. Furthermore, Shin et al. used the same technique to fabricate 3D porous scaffolds based on poly(propylene fumarate)/diethyl fumarate (PPF/DEF). SEM micrographs displayed in Figure 3B reveal the lattice structure of the fabricated scaffold having a line width of 130–160 µm and a pore size of 330–360 µm suitable for tissue engineering applications. The pore size falls within the optimal range for bone tissue engineering, facilitating cell infiltration, nutrient transport, and vascularization. The uniformity in structure and dimensions indicates good control over the fabrication process, likely contributing to reproducible mechanical and biological performance [69]. Since the synthesized materials present a hydrophobic surface, which has a negative effect on cell adhesion, proliferation, and differentiation, the authors used three different peptides (RGD, cyclo RGD, and RGD-KRSR mixture) to modify the surface properties of the material. The effect on MC3T3-EI pre-osteoblast cells has been tested for all formulations [69]. PPF/DEF scaffolds modified with bioactive peptides demonstrated significantly improved initial adhesion and proliferation of MC3T3-E1 pre-osteoblasts compared to unmodified scaffolds. The combination of RGD and KRSR peptides showed the most pronounced effects. This may be due to the presence of KRSR, which can also be found in bones, where it is related to adhesive proteins and enhances proteoglycan-mediated osteoblast adhesion. The RGD-KRSR peptide mixture was more effective than other individual peptide modifications in promoting both early cell attachment and sustained proliferation, likely due to the synergistic effects of integrin-mediated (RGD) and proteoglycan-mediated (KRSR) adhesion mechanisms. Higher concentrations of RGD peptide further enhanced initial cell attachment and proliferation, but this effect plateaued after 7 days of culture, indicating a time-limited influence on proliferation. ALP activity, a marker of osteogenic differentiation, was initially lower in peptide-modified scaffolds but significantly increased after 14 days, particularly in scaffolds modified with the RGD-KRSR mixture. This suggests a delayed but enhanced differentiation response [69].

Luo and colab. synthesized PPF-based oligomers through ring opening polymerization of maleic anhydride and propylene oxide. This method proved to be appropriate for the development of PPF-based resins suitable for 3D printing due to the low viscosity (range between 0.0288 ± 0.0009 dL/g to 0.0780 ± 0.0022 dL/g), molecular mass distribution (Đm), and well-defined molecular mass that can be achieved. Additionally, due to these characteristics, the use of solvent to modulate material flow during 3D printing is considerably decreased. The synthesized oligomers were successfully used to obtain scaffolds that were further evaluated in terms of cytotoxicity. The cytotoxicity of the PPF thin films was assessed using a direct contact test with testing periods at 24, 48, and 72 h [13]. The fluorescence microscopy results from the live/dead assay indicated near-quantitative viability of cells, with no signs of toxicity observed, suggesting that the PPF thin films are biocompatible and nontoxic. The results of standardized test demonstrate that the 3D-printed materials are safe for human mesenchymal stem cells and L929 mouse fibroblasts [70].

Digital light processing (DLP), also called dynamic mask photolithography, is another method of 3D printing that uses a UV or visible light projector to selectively crosslink a photosensitive resin layer by layer [71]. The DLP printing process is characterized by high printing speed than other 3D printing techniques, such as stereolithography (SLA) or fused deposition modeling (FDM), and also has a lateral resolution at 10−100 μm scales [72]. Table 3 summarizes the main differences between SLA, DLP, and FDM [73,74,75].

**Figure 4:**
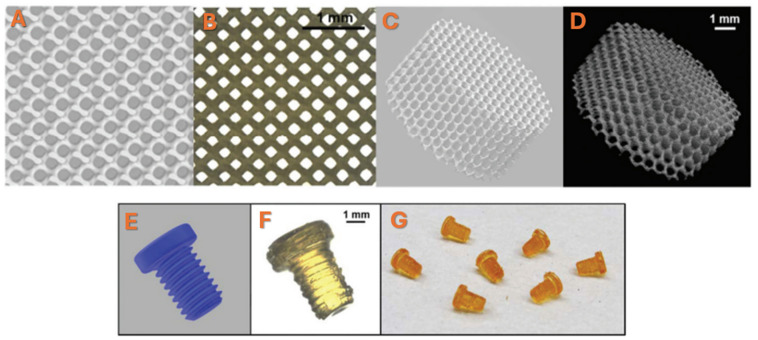
(**A**,**C**) CAD model of a gyroidal scaffold with 136 μm strut size, 475 μm pore size, and 88.2% porosity. (**B**) Optical microscopy image and (**D**) μ-CT image of a 3D-printed gyroidal scaffold (8 mm × 4 mm, porosity: 87.6%, strut size: 130.9 ± 9.3 µm, pore size: 442 µm). (**E**) CAD model of a screw with 4.5 mm height. (**F**) Optical microscopy image of a 3D-printed screw based on PPF. (**G**) Picture of several screw. Reprinted with permission from Ref. [78].

In DLP-based 3D printing, keeping the viscosity of the resin low is essential to avoid failures [76,77]. This technique was used by Le Fer et al. to synthesize a star-shaped PPF molecule using sugar-based alcohol meso-erythritol as initiator. The novel architecture of the designed PPF moiety endows the use of this polymer for DLP 3D printing technique, even though its molecular weight was almost eight times higher in comparison with the largest PPF oligomer previously used in 3D printing. Thus, the authors successfully demonstrated that reduced viscosity (by using DEF) can accompany high molecular mass and evidence the possibility of using such molecules for the printing of complex scaffolds and constructs with high functionality and fidelity, achieving at the same time superior mechanical resistance [78]. Better adhesion of the gyroid structures on the basement plate was made possible by a 25 μm layer thickness, and the curing time/layer was significantly reduced when the total PPF: DEF (DP) of the PPF employed in the formulation was increased. Optical microscopy and X-ray micro-computed tomography (μ-CT) was used to characterize the scaffolds in order to ascertain their true strut size and total porosity and to show the gyroid design elements in more detail (Figure 4A–D). The reported values were around 88% porosity, 450 µm pore size, and 133 µm strut size. Furthermore, using a CAD model, the PPF star-based screws were printed at a high of 4.5 mm, and the optical images of the screws can be seen in Figure 4E–G.

Luo et al. [79] assessed in their study the influence of several parameters for the DLP 3D printing process by employing several PPF oligomers as resin. The oligomers were synthesized using a ring-opening copolymerization of two compounds, including maleic anhydride and propylene oxide, and then isomerization with a base. Viscosity, polymer concentration, degree of polymerization, and resin printing temperature proved to have a strong influence over the processing method. The mechanical tests performed on the printed scaffolds demonstrated good tensile properties (from 178 ± 38 MPa to 199 ± 8 MPa), which are similar to the human trabecular bone [79].

**Figure 5:**
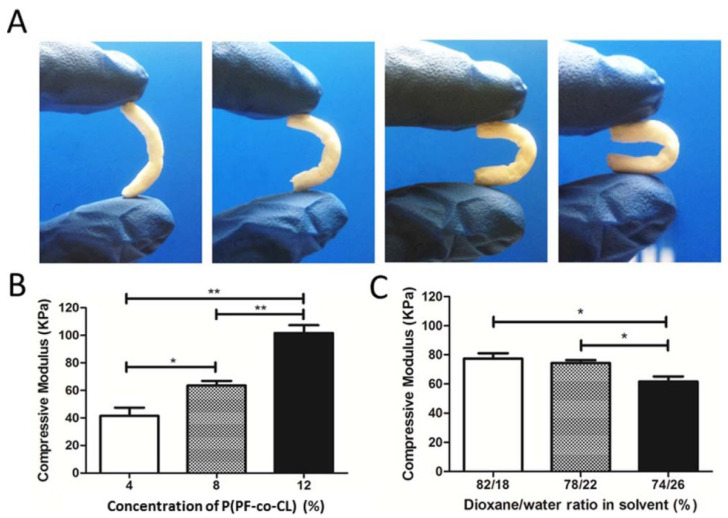
(**A**) Poly(propylene fumarate-co-caprolactone) [P(PF-co-CL)] scaffold samples (8% polymer concentration and 76/24 dioxane-water ratio solvent) subjected to flexural stress. (**B**) Compressive modulus of P(PF-co-CL) scaffolds (in a dioxane–water solution of 76/24 wt/wt). (**C**) Compressive modulus of 10-wt%. P(PF-co-CL) copolymer scaffolds as a function of different solvent ratios with * *p* < 0.05; ** *p* < 0.01. Reprinted with permission from Ref. [82].

Thermally induced phase separation (TIPS) represents another valuable technique that can be used for the synthesis of porous scaffolds with controlled architecture and interconnected cavities. This technique is based on phase separation of a polymeric solution by decreasing the temperature. Upon cooling, a polymer-rich phase and a polymer-lean phase are obtained, which can further on be converted into scaffolds by removing the solvent through extraction, evaporation, or sublimation [80,81]. Guo et al. used a ternary system (74/26, 78/22, and 82/18 wt./wt. ratio) comprising poly(propylene fumarate-co-caprolactone) P(PFco-CL) dioxane, and water to fabricate porous scaffolds through TIPS. As can be observed from Figure 5, the compressive modulus of the scaffolds increased with higher polymer concentration and greater dioxane content in the solvent mixture. Specifically, scaffolds with 12% P(PF-co-CL) and a solvent ratio of 82/18 (dioxane/water) demonstrated the highest mechanical strength. Meanwhile, the bending test images (Panel A) show that the scaffolds maintain notable flexibility, indicating a balance between strength and elasticity. These properties suggest strong potential for applications in cartilage tissue engineering and nerve regeneration, where both mechanical support and flexibility are essential.

The fabrication of the porous structures is greatly influenced by the ratio of solvents to polymer concentration, as demonstrated by the results, and the ideal processing parameters were determined to be 22 wt.% to 24 wt.% water content and 8 wt.% polymer concentration [82].

## 3. Synthesis of Bone Tissue Engineering Composite Scaffolds Based on PPF

PPF has been extensively studied for the synthesis of functional constructs for bone repair as both prefabricated scaffolds or as injectable in situ curing material [83,84]. Despite its numerous advantages, PPF still has a number of limitations mostly coming from weak inherent mechanical properties that make it inappropriate for use in load-bearing orthopedic applications. Apart from that, the high viscosity at room temperature makes it difficult to handle and also limits its processability through novel fabrication techniques such as 3D printing. A possible solution for these problems would be the use of blends with other polymers and copolymers or the use of reinforcing agents to develop advanced composite formulations.

Up to now, PPF has been used along with different inorganic substances, such as titania [85], calcium phosphates [86], hydroxyapatite (HA) [87], calcium carbonate, or sulfate [88], or carbonaceous materials, which are known for their osteoconductive and mechanical improvement effect. The same inorganic materials were also used as reinforcing agents for other kind of polymers. For example, titanium dioxide (TiO_2_) or silicon dioxide (SiO_2_) enhances the mechanical parameters like strength and fatigue resistance of an epoxy resin, while graphene or graphene oxide opens up new possibilities in electronics and other cutting-edge technologies by significantly increasing electrical conductivity and resilience to heat shocks [89].

*Inorganic Substances*

**Figure 6:**
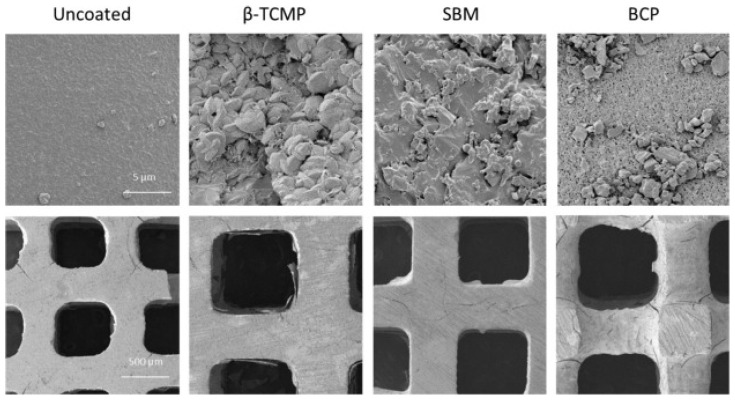
SEM micrographs displaying the morphology and roughness of PPF scaffolds coated with different calcium phosphates. Reprinted with permission from Ref. [57].

Dadsetan et al. [57] assessed in their study the influence of calcium phosphate coating and concurrent administration of recombinant human bone morphogenetic protein-2 (rhBMP-2) over the in vivo bone regeneration potential of biodegradable, porous poly(propylene fumarate) scaffolds. SEM micrographs (Figure 6) revealed that the presence of the inorganic compound does not alter the porosity of the materials. Moreover, it seems that the scaffolds covered with calcium phosphate may continue to produce rhBMP-2, while an open structure of internal porosity is maintained. According to mechanical testing of the flaws, the synthetic bone mineral and b-tricalcium phosphate showed strength restoration following rhBMP-2 administration [57].

In the work reported by Qayoom et al. [60], PPF and nanohydroxyapatite (Nhap) scaffolds were synthesized by light-induced crosslinking. The synthesized composite materials were used as dual drug delivery vehicles for ciprofloxacin (CIP) and rifampicin (RFP) as potential treatment for bone infections. The two therapeutic agents were gradually integrated within the formulation by first adding the CIP alongside PPF and 40% nHAP. The second antibiotic was included by dipping the previously obtained scaffold in a gelatin solution containing rifampicin, leading to ciprofloxacin-loaded scaffolds coated with rifampicin (PHRC). In vitro antibiotic release study revealed that after 60 days, PHRC demonstrated a complex release behavior with a temporal separation between the two drugs loaded. Thus, RFP was the first one released over an interval of 7 days with a cumulative release of 29.72 ± 0.0034 μg/mL followed by CIP, wherein a cumulative release of 62.43 ± 0.21% was noted for a period of 60 days [60].

**Figure 7:**
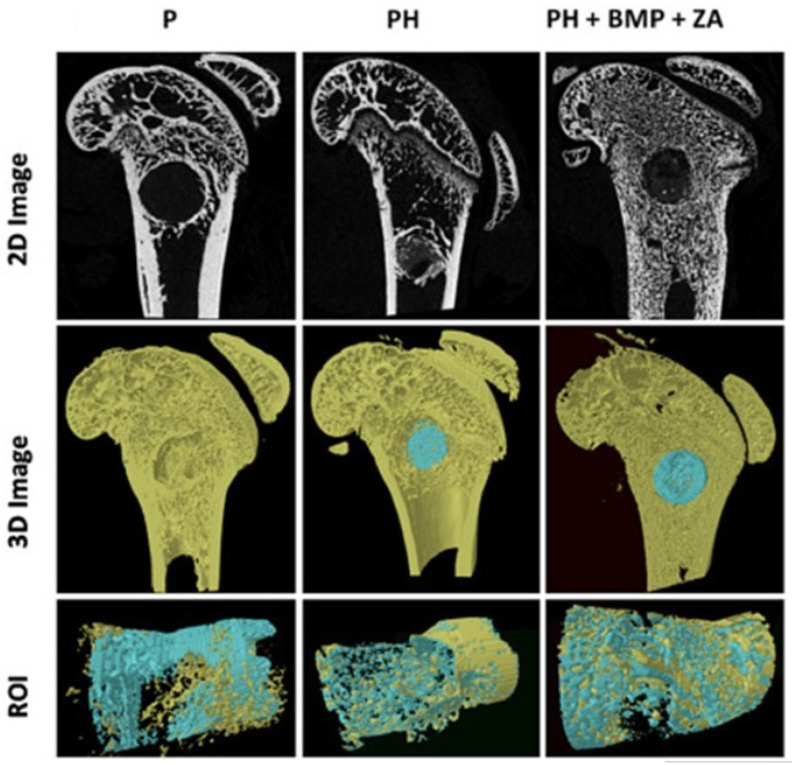
2D and 3D micro-CT scans revealing bone formation in rat femoral condyle fracture model in PH + BMP + ZA group and almost-empty defect in P group. The 3D image of ROI selected shows complete filling of the defect and dense bone formation in PH + BMP + ZA group. The old bone is represented with yellow while the newly formed bone with blue. Reprinted with the permission from Ref. [60].

An in vivo infection model was also used to analyze the ability of the scaffold to lower the bacterial burden at the infection site. The results of this study indicated that RFP-coated, CIP-loaded PPF composite scaffolds may lessen bacterial load while also promoting bone repair in infection-related conditions [60]. Furthermore, the inorganic-organic composite scaffolds’ ability to promote fracture healing in a femoral condyle fracture model has been illustrated in the micro-CT images (Figure 7). They observed that the sample composed of polypropylene fumarate-nanohydroxyapatite composite + bone morphogenetic protein + zoledronic acid (PH + BMP + ZA) (6.25 ± 0.6 mm^3^) presents good enhancement of the bone mineralization in comparison with polypropylene fumarate (P) (2.61 ± 1.0 mm^3^) sample and PH alone (2.54 ± 0.8 mm^3^) after 4 weeks, leading to improved bone growth.

Furthermore, PPF was used by Teng et al. to synthesize nanocomposite materials based on different concentrations of hydroxyapatite (HA) as viable substitutes for cervical cages [87]. The ability of the proposed formulation to promote trabecular bone formation was harvested by achieving a porous structure through a salt-leaching technique. Mechanical tests revealed that HA had a significant beneficial effect on the mechanical properties of the materials. A 20 wt.% HA loading improved the hardness by 25% in comparison with pure PPF matrix while stiffness increased by 85%. The observed findings imply that this polymeric composite may make an excellent candidate as a material to develop an intervertebral body cage.

Wang et al. [90] assessed the potential of a novel complex nanocomposite formulation based on hydroxyapatite (HAP), PPF, and poly(propylene fumarate) diacrylate (PPF-DA) to be used as injectable bone cement. The potential of the nanocomposite systems to be used as an alternative for polymethyl methacrylate in the orthopaedic field was evaluated. Mechanical and degradability tests demonstrated that both the HAP content and double bond content have a strong influence. Thus, lap-shear and bonding strength results revealed that 3% wt. composite led to better properties (0.71 and 5.24 MPa) as a consequence of higher crosslinking density achieved at a lower C=C bond concentration. In vitro degradation tests showed that the degradation rate of nanocomposite formulations exhibited a trend that corresponds with the generation rate of new bone, thus highlighting their potential as tissue engineering materials. Apart from that, biocompatibility tests on MC3T3-E1 cells revealed that the composite materials do not induce cell death and lead to proliferation and adhesion of the cells [90].

Trachtenberg et al. [91] synthesized 3D scaffolds based on PPF/ceramic composite with HA gradients using extrusion-based printing techniques. HA nanoparticles used as nanoreinforcing agents were included in the formulations in order to emulate the natural composition of bone tissue. The authors successfully combined the advantages of artificial, biodegradable PPF polymer with the strong compressive mechanical characteristics of osteoconductive HA nanoparticles. The final 3D-printed scaffolds had clearly defined layers with interconnected pores [91].

**Figure 8:**
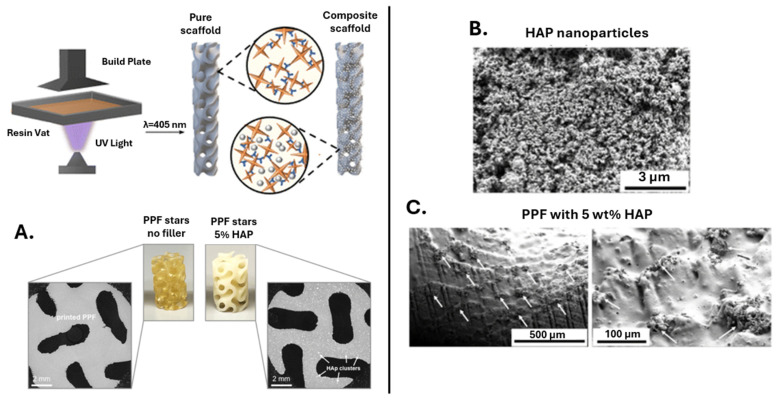
Schematic representation of 3D printing process for PPF hydroxyapatite nanocomposite scaffolds along with μCT scans (**A**), SEM micrographs displaying the uniform distribution of the Hap nanoparticles (**B**), and individual HAp nanoparticles and aggregates present on the surface of the printed structures (**C**). Reprinted with permission from Ref. [92].

A recent study analyzes the influence of the post-printing processing conditions of nanocomposite scaffolds based on four-arm stars PPF polymer-reinforced with 5 wt.% HA on the mechanical performance (Figure 8) [92]. The viscosity of the PPF was modulated by adding ethyl acetate as a solvent and was measured by rheological tests. The reinforcement effect of HA nanoparticles was evidenced through dynamic mechanical analysis. The difference in Tg recorded for the two samples with different post-processing conditions is approximately 20 °C. This difference may be associated with obtaining a strong and compact network after 6 days of drying at 60 °C. The authors successfully demonstrate that without proper post-processing conditions, the mechanical properties of the materials considerably decrease from ~320 MPa to ~74 MPa for the matrix and from ~325 MPa to ~79 MPa for the nanocomposites.

**Figure 9:**
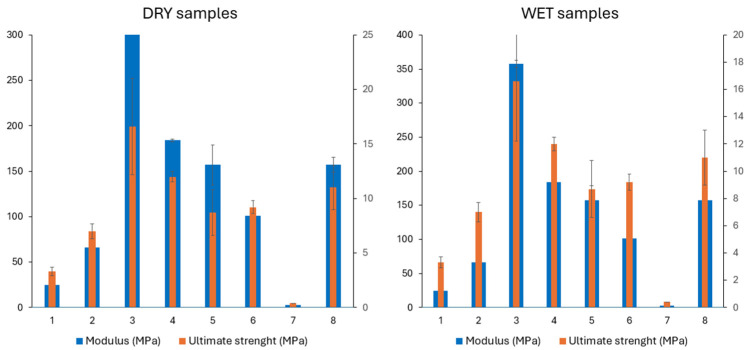
Comparative analysis of ultimate strength and compressive modulus for nanocomposite hydrogels based on results from [71].

Nagwa et al. [93] proposed N-vinyl pyrrolidone (NVP), methyl methacrylate (MMA), and a combination of NVP and MMA (1:1 weight ratio) as crosslinking agents to synthesize nanocomposite materials based on PPF/HA. Different concentrations of natural bone powder (5, 10, and 15% wt.) and 45 wt.% HA have been used for the synthesis of composite materials [93]. The presence of natural bone powder proved to have a positive effect on the compressive strength, reaching a value of 139.89, 127.66, and 65.82 MPa at strain 0.36% for the composite loaded with 10 wt.% natural bone powder and crosslinked with NVP, NVP/MMA, and MMA. Zhu et al. used bone fibers to synthesize composite formulation based on PPF crosslinked with NVP [94] and evaluated the mechanical properties as potential biomaterials in orthopaedics. The authors performed a thorough evaluation of the multiple factors that may influence the final properties of the materials, such as the type of bone fibers (either mineralized or demineralized), molecular weight of PPF and the concentration of crosslinking agent (NVP) used, initiator (benzoyl peroxide BP), and porogen agent (sodium chloride). They observed that the compressive modulus of the dry and wet samples ranged in absolute values from 21.3 to 271 MPa and 2.8 to 358 MPa, respectively, while the ultimate strengths ranged from 0.4 to 16.6 MPa for wet samples and from 2.1 to 20.3 MPa in the case of the dry samples, as presented in Figure 9.

The best results were obtained for the samples synthesized with mineralized bone fibers and the highest concentration of BP, while the molecular weight of PPF had no significant influence over the properties [94].

**Figure 10:**
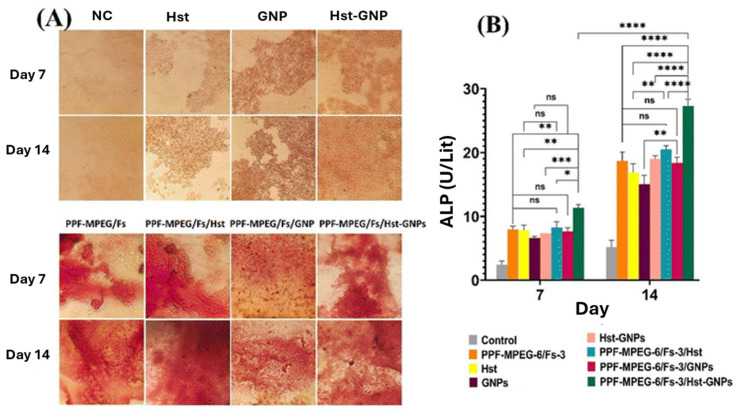
The in vitro osteogenic activity of studied hydrogels by (**A**) the alizarin red staining test and (**B**) alkaline phosphatase assay after 7 and 14 days of incubation with MG-63 cells (ns: non-significant, (* *p* ≤ 0.05, ** *p* ≤ 0.01, *** *p* ≤ 0.001, **** *p* ≤ 0.0001)). Reprinted with the permission from Ref. [95].

In the study developed by Zaghian et al. [95], forsterite (Fs) was used to enhance the osteoconductivity of PPF/methoxy polyethylene glycol injectable hydrogel. Gliadin nanoparticles loaded with hesperetin flavonoid (Hst-GNPs) were also included in the formulations, and their influence on biological properties was assessed. The presence of the Hst–GNPs exhibit a noticeable improvement in cell proliferation, differentiation, and mineralization, as demonstrated by in vitro osteogenic activity tests (Figure 10). Alizarin red staining (Figure 10A) demonstrates that the PPF–MPEG-6/Fs-3 hydrogel incorporating Hst–GNPs significantly enhances osteogenic activity, as evidenced by increased calcium deposition over 7 and 14 days. The synergistic effect of Hst–GNPs and the bioactive hydrogel matrix promotes greater mineralization compared to individual components, highlighting its potential for bone tissue engineering applications. Both PPF–MPEG and PPF–MPEG/Fs scaffolds showed some degree of mineralization when immersed in simulated body fluid, indicating that both materials have the potential for bone tissue engineering applications. However, PPF–MPEG/Fs scaffold exhibited a higher degree of mineralization compared to the PPF–MPEG scaffold, suggesting that the addition of Fs enhances the biomineralization properties of the hydrogel. The PPF–MPEG-6/Fs-3/Hst–GNP hydrogel significantly increased ALP activity, a marker for osteoblast differentiation, compared to various control groups after 7 and 14 days of cell culture (Figure 10B). This enhanced activity and mineralization were due to the synergistic effects of the PPF–MPEG/Fs hydrogel and Hst–GNPs, promoting osteogenesis. These results highlight the potential of this hydrogel in promoting osteointegration for bone tissue engineering [95].

A sintered microsphere approach was used by Shahabi and colab. to create degradable poly(propylene fumarate)/bioactive glass (PPF/BG) composite scaffolds [39]. In vitro bioactivity of the composite scaffold was evaluated by immersion in simulated body fluid. SEM analysis revealed the formation of a thin layer of hydroxycarbonate apatite (HCA) that almost covered the surface of the scaffolds after 14 days. In vitro degradation tests revealed that after 12 weeks, both the matrix and the composite lose around 10–12% of their weight. The presence of bioactive glass within the composite scaffolds did not influence the mechanical properties, which remained constant during the 12 weeks of in vitro degradation [39].

Boron nitride nanotube (BNNT) novel 1D nanomaterials have recently drawn researchers’ attention as reinforcing agents due to their exceptional physical properties. In the article reported by Diez-Pascual [96], chemical vapor deposition (CVD) was used to synthesize inorganic boron nitride nanotubes, which were then integrated into a biocompatible PPF matrix by ultrasonication in ratios between 0.1–5% [96]. Mechanical tests revealed that the Young’s modulus gradually increased from 1 GPa recorded for the matrix to 8 GPa for 5% nanocomposite, revealing that the presence of BNNT has a significant influence on the mechanical properties. This may be a consequence of higher compatibility between the components of the systems achieved through hydrogen bonds and Van der Waals interactions. Thus, the authors successfully demonstrate the potential of BNNT that, in this case, surpasses the performance of single-walled carbon nanotubes (SWCNTs) as reinforcing agents to be used in the synthesis of biomaterials for orthopedic scaffolds [96].

**Figure 11:**
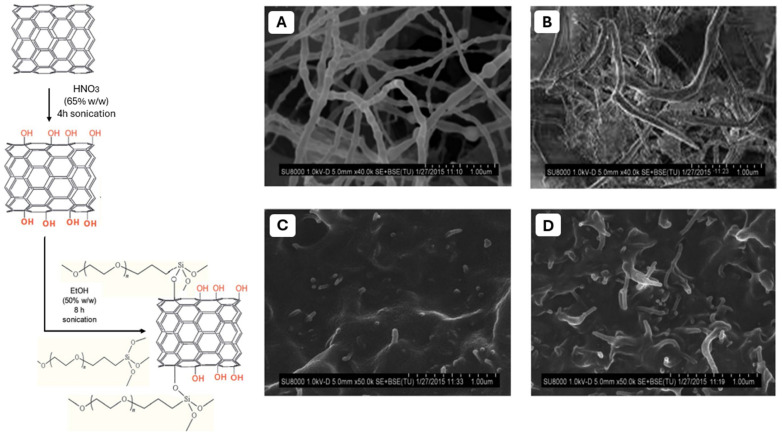
Reaction pathway for the synthesis of PEGylated BNNT nanotubes. SEM micrographs of raw BNNTs (**A**), PEG-g-BNNTs (**B**), PPF/PEG-g-BNNTs (0.5 wt%) (**C**), and PPF/PEG-g-BNNTs (4.0 wt%) (**D**). Reprinted with the permission from Ref. [97].

In order to increase the compatibility between PPF and BNNT, PEGylation of the nanotubes was proposed by Díez-Pascual et al. [97]. The modification of BNNT was performed by grafting silane-PEG on the surface of the nanotubes. SEM results indicated a homogeneous distribution of the PEG-grafted BNNTs within the PPF matrix at lower filler concentration, which leads to good compatibility between the covalently functionalized nanotubes and the matrix (Figure 11). Improved polymer matrices-filler interfacial adhesion and prevention of nanofiller aggregation should result from the polar and hydrogen bonding interactions between the ester moieties of PPF and the ether and hydroxyl groups of PEG-g-BNNTs. This would lead to better nanocomposite properties. By increasing the PEG-g-BNNTs amount to 4 wt.%, the structure of the nanotubes becomes more coiled, twisted, and bundled. The nanocomposites outperformed PPF in terms of stiffness and strength, and they were sufficiently rigid under physiological circumstances to be applied as regeneration scaffolds for bone tissue. When PEG-g-BNNTs were added, the authors obtained an increase of the modulus and tensile strength of 2.3-fold enhancement, and the maximum σ_y_ increases by 70% compared with pure PPF. Furthermore, considering the elongation at break, the pure PPF registered low ductility, which tends to drop by 30% after the addition of PEG-g-BNNTs [97].

Karfarma et al. [98] developed a composite material consisting of micro- and nanosized magnesium calcium phosphate (MCP) particles and PPF. The influence of the size of the particles on the mechanical properties, along with the concentration (5, 10, 20%) of the filler, were evaluated for the synthesized scaffolds. Compressive strength and modulus analysis revealed a significant improvement in these properties, regardless of the dimensions of the MCP particles. Thereby, in the case of the 5 wt.% MCP, there was a difference of ~2 MPa between the different size nanofillers. However, the best mechanical properties were recorded for 10 wt.% MCP nanocomposite, achieving a Young modulus of ~33 MPa and the compressive strength ~420 MPa. At higher concentrations of 20 wt.% MCP, both micro- and nanoparticles tend to agglomerate and thus lead to the formation of structural defects that decrease the toughness of the composite scaffolds [98].

Tungsten disulfide nanotubes (WSNTs) were used by Lalwani et al. as reinforcing agents for PPF-based nanocomposites as bone tissue materials. The efficiency of the inorganic tungsten nanotubes was compared with single- and multi-walled carbon nanotubes. The obtained results show that the mechanical reinforcement exhibited by inorganic nanotubes (WSNTs) was either comparable to or superior to that of carbon nanotubes (SWCNTs and MWCNTs), with results reaching up to 127%. However, WSNTs showed a positive impact over the crosslinking density of the PPF matrix due to the presence of sulfide and oxysulfide functionalities on their surface [99].

*Carbonaceous Nanostructures*

Carbonaceous nanostructures such as carbon nanotubes (CNT), functionalized graphene oxide (fGO), graphene oxide nanoribbons (GONr) or nanoplatelets, and fullerenes have proved to be valuable nanoreinforcing agents for bone tissue scaffolds based on PPF due to their positive effect on mechanical strength and electrical conductivity. Such properties can lead to the development of a microstructure closer to the anatomy of the natural bone where electrical signalling is present.

**Figure 12:**
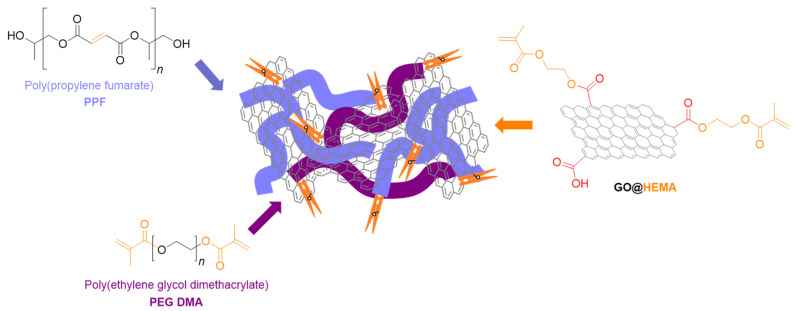
Schematic representation of the PPF/PEGDMA/GO@HEMA composite scaffolds synthesis. Reprinted with the permission from Ref. [100].

Graphene oxide was chemically modified with 2-hydroxyethyl methacrylate (GO@HEMA) by Vasile et al. [100] to develop novel reinforcing agents for poly(propylene fumarate)/poly(ethylene glycol) dimethacrylate (PPF/PEGDMA) for the synthesis of advanced nanocomposite materials for bone tissue engineering (Figure 12). When 1 wt.% GO@HEMA was added in the PPF/PEFDMA matrix, the compressive modulus was 14-fold higher than the matrix. However, when 2% of GO@HEMA was used, the compressive modulus dramatically decreased as a consequence of agglomeration tendency of the nanofiller, an aspect sustained also by SEM analysis. In vitro tests demonstrated that the synthesized nanocomposites have no cytotoxic effect and also have a high biocompatibility with murine pre-osteoblasts [100].

**Figure 13:**
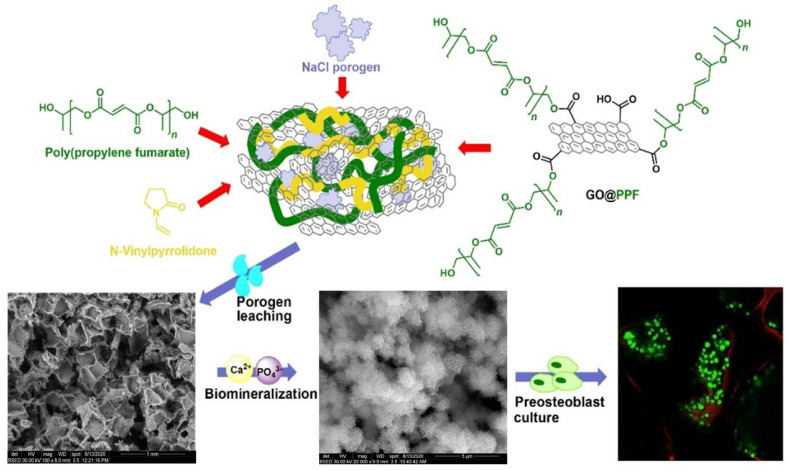
Schematic representation of the porous PPF/NVP/GO@PPF composite scaffolds. Reprinted with the permission from Ref. [101].

In a further study comprising a similar approach, Pandele et al. modified the surface of graphene oxide with PPF (GO@PPF) for further use as a reinforcing agent along with poly(propylene fumarate)/*N*-vinyl pyrrolidone(PPF/NVP) [101]. Similar results were also observed for this study in terms of the improved mechanical properties (two-fold improvement) and biocompatibility (Figure 13). The obtained materials display a network with open and interconnected pores, which promote mineralization at their surface and a good interaction between PPF/PVP/GO@PPF and murine pre-osteoblasts in terms of viability, proliferation, and adhesion.

Díez-Pascual synthesized PPF-based nanocomposites containing different concentrations of polyethylene glycol functionalized graphene oxide (PEG-GO) [102]. As a processing method, sonication was used for the dispersion of the nanofiller within the polymeric matrix followed by thermal curing. The mechanical, viscoelastic, antibacterial, and hydrophilic properties of the final materials, as well as surface morphology and structure, were evaluated. Tensile testing indicated that the composites had better stiffness, strength, and toughness in comparison with neat PPF, showing an increase of the modulus and tensile strength from 40 MPa and 25% to 56 MPa and 38% in the case of the composite with the highest PEG-GO loading. Furthermore, even under biological conditions, the nanocomposites maintained sufficient stiffness and strength to effectively assist the formation of bone tissue [102].

Lalwani et al. [103] studied the influence of different 2D nanofillers such as graphene oxide nanoribbons (SWGONRs and MWGONRs), molybdenum disulfide nanoplatelets (MSNPs), and graphene oxide nanoplatelets (GONPs) at 0.01−0.2 wt.% concentrations as reinforcing agents to PPF composites [103]. When the compressive modulus of samples was analyzed, MSNP/PPF sample with 0.2 wt% concentration showed the highest value of ~2100 MPa. This result demonstrated an increase in the mechanical properties in comparison with PPF of 108%. Shi and colab. used single-walled carbon nanotubes (SWNTs) for the synthesis of injectable PPF composites for bone tissue engineering. The authors evaluated the influence of different types of SWNTs, covalently functionalized SWNTs (F-SWNTs) and SWNTs modified with a surfactant over the rheological properties of the unpolymerized composites and mechanical properties of the final materials [104]. For the nanocomposite synthesis, various nanotubes concentrations (0.01–0.2 wt.%) were integrated within the PPF matrix through ultrasonication. Mechanical tests revealed that all types of SWNTs used had a similar effect on the mechanical properties of the final composites with significant improvement in the case of 0.02 wt.% and 0.1 wt.% samples [81]. Furthermore, in another study, the same team reported the in vitro cytotoxicity of the injectable PPF/SWNTs composite using a fibroblast cell line, and the obtained data shows excellent cytocompatibility for use as scaffolds in an application involving bone tissue engineering [105].

The cytotoxicity of poly(propylene fumarate) nanocomposites reinforced with various carbon nanomaterials, manufactured for bone tissue engineering applications, was examined in the study reported by Farshid and colab. [106]. The findings suggest that PPF nanocomposites do not cause acute cytotoxicity at any concentration of nanomaterial loading. Whereas PPF nanocomposites containing 0.1 wt.% GONP are slightly harmful (~82% viability), PPF nanocomposites containing 0.1 wt.% MWGONRs exhibit negligible cytotoxicity (~97% viability).

## 4. Future Directions Integrating AI

**Figure 14:**
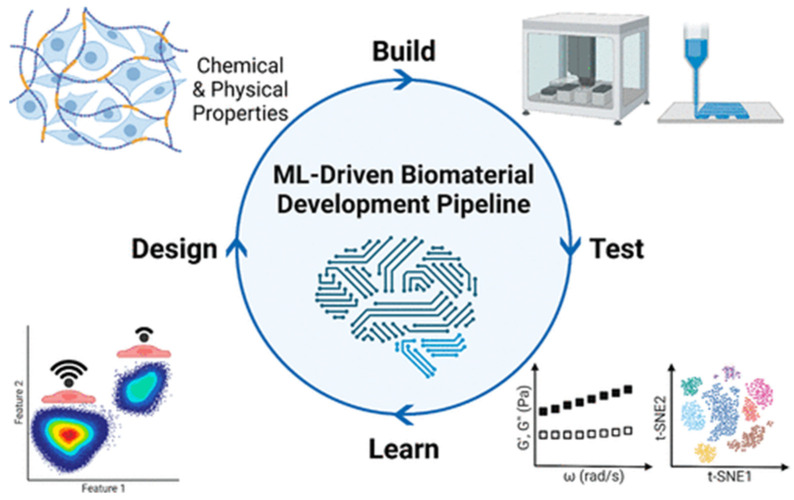
Diagram illustrating an example of the design-build-test-learn framework for biomaterials development. Reprinted with the permission from Ref. [108].

In recent years, the advancement of artificial intelligence (AI) has transformed healthcare and is essential in many fields, including predicting and diagnosis. The amount of biological data has grown significantly over the past 10 years, and medical technology development has seized the chances presented by the computer age. Throughout the various phases of TE, AI can be applied in a variety of ways that could not only overcome current constraints but also speed up procedures, improve accuracy and efficiency, lower costs, and lessen post-transplant problems. Using the finite element method, the mechanical properties of the scaffold material may be predicted while considering its microarchitecture and matrix material properties. These methods can boost the productivity and efficiency of creating scaffolds with special qualities for the final user while also drastically lowering the costs of time and resources. In the synthesis and manufacturing of skeletons, AI enables the development of trustworthy models that have the potential to support the regeneration of living tissues. As seen in Figure 14, the advent of artificial intelligence technologies presents numerous prospects for the creation of a multipurpose material that satisfies the different requirements of tissue engineering, including favorable chemical composition, density, adhesive surface, and biological activity [107].

The design-build-test-learn (DBTL) model represents a powerful and systematic framework that can be employed to enhance the development of advanced biomaterials. In this approach, materials are initially designed based on rational design principles considering chemical and physical features. These designs aim to target specific mechanical, chemical, or biological functionalities. Once designed, materials are built, typically through methods such as polymer synthesis or additive manufacturing, and subsequently tested for desirable properties using high-throughput and automated laboratory techniques. The resulting experimental data, coupled with initial design parameters, are then analyzed in the learn phase using machine learning (ML) algorithms. These algorithms extract meaningful patterns and generate predictive models that reveal key relationships between material structure and performance. These models can then be exploited to tailor the design of new materials with optimized or targeted functionality, thus completing the cycle. This iterative, data-driven workflow not only enhances efficiency and precision in materials research but also plays a key role in developing next-generation biomaterials for applications such as tissue engineering, regenerative medicine, and personalized therapeutic strategies.

**Figure 15:**
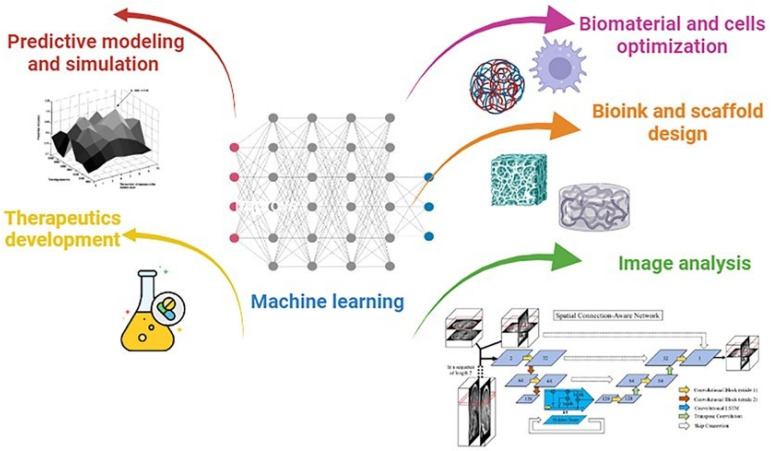
Schematic representation of the main operations and parameters that can be assisted by machine learning. Reprinted with the permission from Ref. [109].

Additionally, the use of AI along with machine learning (ML) and deep learning (DL) can lead to significant improvement (Figure 15). For example, AI can create more functional tissues and tailor medical devices while boosting productivity at the same time since ML employs algorithms that are intended to identify patterns, learn about the surroundings, and offer solutions. On the other hand, neural networks (NNs) are used in DL in cell culture to improve the scaffold design for target tissues. Finding patterns in big datasets can also help with this, as it can streamline feature identification and cut down on the number of experiments required to produce a structure [109].

Furthermore, the overall effectiveness of TE projects could be greatly increased by using AI techniques and ML. ML has several important applications in tissue engineering, such as improving drug delivery systems, predicting material–cell interactions, aiding image analysis, modeling the performance of in vivo scaffolds, and enhancing bioprinting and other scaffold design procedures [110]. ML provides the groundbreaking potential to enhance scaffold design by the methodical examination of the relationship between biological reaction, material qualities, and fabrication parameters.

It is not just challenging but also time- and resource-intensive to design and solve TE problems since tissues and organs are complex systems that need multiple approaches for proper development. In order to identify patterns, learn about the ideal environment of a specific tissue, and propose solutions, ML uses algorithms that are capable of identifying particular characteristics in specific datasets that may further facilitate a complex and elevated comprehension of molecular or cellular processes. Up to now, AI has been used in numerous medical-related domains such as drug delivery, radiology, preclinical models for cancer study, diagnostic, and personalized treatments [111,112,113,114,115].

**Figure 16:**
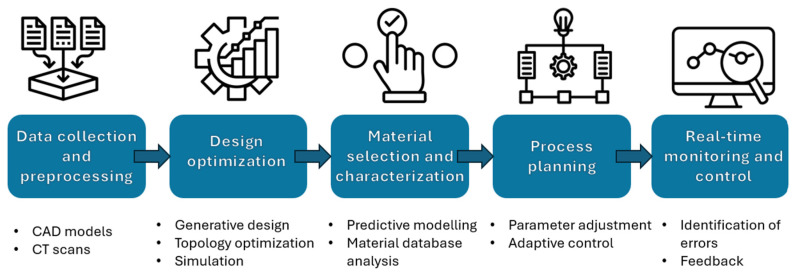
Workflow for the development of a scaffold using AI-integrated 3D printing.

3D printing, the TE’s top manufacturing method at the moment, is a complex dynamic process that requires multiple steps that consequently can generate distinct errors and difficulties, even though proper optimization of both material and parameters was thoroughly established prior printing. In this context, AI represents a powerful tool that not only can prevent mechanical errors related to the printer or temperature fluctuations that can influence the behavior of the material but also to overcome them. Recently, the use of AI has been extensively exploited in 3D printing field, as it is one of the most valuable and efficient technique to replicate and fabricate tissue substitutes. The main steps followed by this process are presented in Figure 16.

**Figure 17:**
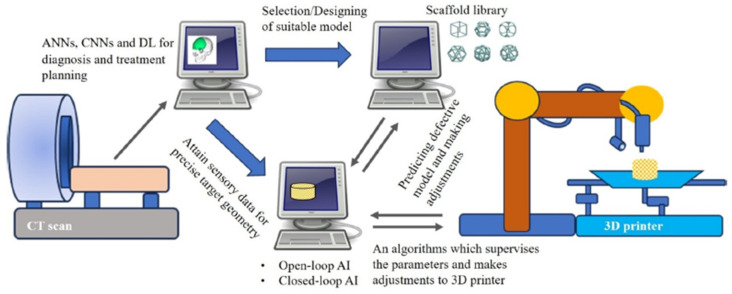
Schematic representation of the main steps involved in the synthesis of a 3D scaffold using artificial intelligence. Reprinted with permission from [117].

Usually, a dataset of images obtained by computed tomography or magnetic resonance imaging is typically needed to design the geometrical model of the scaffold. Moreover, in order to design a scaffold with ideal functionality, the architecture must be projected accordingly with the need to fulfill both mechanical and biological parameters. Complex algorithms are then run to segment the tissue, reconstruct the volume, and generate the 3D printing blueprint [116]. For example, AI and 3D printing technologies were employed by Logeshwaran and colab. to improve bone tissue restoration, offering a more practical and economical remedy for bone-related issues caused by aging, infections, and accidents [117]. By allowing the 3D printing process to anticipate, adjust, and regulate its own parameters, artificial intelligence (AI) can help reduce potential errors (Figure 17).

Also, the electrospinning process holds considerable promise for the widespread use of ML. In 2024 with a binary classification techniques, López-Flores et al. predicted whether or not aligned nanofibers would be produced. They also used regression models and artificial neural networks (ANN) to predict the nanofibers’ orientation, angle, and diameter. ANNs performed quite well, with binary classification accuracy of 0.94 and validation test accuracy of 0.90 [118]. Carotenuto et al. utilized a logical framework based on the design of experiments to produce heuristic models that capture the links between scaffold qualities (Ys) and process parameters (Xs) in order to produce five polycaprolactone scaffolds. A series of characteristics (Ys), including the distribution of fiber diameter, porosity, wettability, Young’s modulus, and adhesion of mouse C1C12 myoblast cells, were measured by characterizing the scaffolds. The study’s findings confirm that statistical mapping of electrospinning processes may be implemented and that TE scaffolds can benefit from the statistical models that are produced [119].

ML was also been used to forecast the creation of self-assembled peptide hydrogels in the work of Li et al. [119]. As a training set for machine learning, the authors produced a library of 2304 compounds made by the Ugi reaction from 31 monomers, comprising 8 amines, 8 aldehydes/ketones, 12 Fmoc-amino acids, and 3 isocyanides. For the random forest, logistic regression, and gradient boosting, the corresponding precisions were 54%, 57%, and 62%. When the dataset’s quality is improved, these indicators most likely may be higher. Data resampling was used since the authors noticed that the data were extremely unbalanced (less than 4% of cases corresponded to hydrogel-forming).

Considering PPF-based scaffolds, the incorporation of AI into their synthesis and characterization has an enormous potential to address present difficulties while driving innovation. AI can transform scaffold design by allowing machine learning (ML) algorithms to anticipate the best topologies for specific tissues based on porosity, mechanical strength, and biodegradability. Advanced technologies such as generative models could investigate novel biomaterials by mining large databases, modelling material properties, and suggesting interesting combinations, eliminating the need for trial-and-error research. Artificial intelligence-assisted fabrication techniques, such as 3D printing and electrospinning, could use adaptive algorithms to improve manufacturing settings in real time, ensuring precision and consistency. AI-powered characterization systems can automate data processing from imaging and spectroscopy techniques, providing more information about scaffold structure and performance. Furthermore, predictive models mimicking scaffold–cell interactions could speed up the design of scaffolds with better biological integration while minimizing in vivo testing.

By following these strategies, researchers can recognize the full potential of AI in tissue engineering, allowing for faster, more precise scaffold construction and characterization. These advancements aim to increase scaffold performance, improve patient outcomes, and bring tissue-engineering technologies closer to clinical use.

## 5. Discussions

This study contributes to the emerging insights into the fabrication of PPF-based scaffolds, focusing on their potential for advanced bone and tissue engineering applications. Through an extensive review and comparative analysis of both conventional and state-of-the-art manufacturing techniques, we highlight how scaffold architecture, material formulation, and fabrication strategy critically impact the structural, mechanical, and biological performance of tissue-engineered constructs.

Conventional fabrication methods such as porogen leaching and gas foaming lack the precision and reproducibility required for clinical applications leading to scaffolds with inconsistent mechanical strength and poor control over degradation, thus limiting their applicability. In contrast, modern additive manufacturing techniques, especially SLA, μSLA, DLP, and FDM, have emerged as promising alternative methods that allow for the precise engineering of scaffolds with customized pore sizes, shapes, and interconnectivity, attributes essential for nutrient diffusion, cellular infiltration, and vascularization.

Among the examined methods, stereolithography (SLA) and its derivatives, such as μSLA and DLP, stand out due to their high spatial resolution and compatibility with photocurable polymers such as PPF. The studies analyzed collectively demonstrate that PPF’s mechanical and degradation properties can be finely tuned through molecular weight, crosslinking density, resin viscosity (via DEF modulation), and printing parameters. Moreover, the printability and structural fidelity of PPF-based scaffolds have improved through novel fabrication methods such as extrusion-based 3D printing. The ability to create scaffolds with HA gradients and interconnected pores allows for better mimicry of natural bone architecture. Additionally, studies underscore the importance of post-processing conditions on the final mechanical properties, where insufficient curing dramatically reduces performance, indicating that scaffold manufacturing parameters must be carefully optimized.

Further, the importance of surface bioactivity was underscored through the functionalization of PPF/DEF scaffolds with peptides like RGD and KRSR. These modifications significantly enhanced early cell adhesion, proliferation, and osteogenic differentiation, confirming the role of surface chemistry in scaffold performance.

The increasing demand for bioactive, mechanically stable scaffolds for bone tissue engineering has driven the advancement of PPF-based composite systems. Despite its excellent biocompatibility and in situ polymerizable behavior, PPF exhibits inherent limitations such as relatively weak mechanical properties and high viscosity at room temperature. Inorganic reinforcement has proven to be a critical strategy in overcoming PPF’s limitations. A variety of materials, such as hydroxyapatite (HA), calcium phosphates, titania, and bioactive glass, have demonstrated significant improvements in compressive strength, stiffness, and osteoconductivity. For instance, the incorporation of nanohydroxyapatite or calcium phosphate not only strengthened PPF scaffolds but also enabled dual drug delivery systems that effectively supported bone regeneration while combating infection, as demonstrated by Qayoom et al. [60]. These systems showed prolonged and sequential antibiotic release, highlighting the versatility of PPF composites as both structural and therapeutic platforms.

The chemical modification of reinforcing agents, such as PEGylated boron nitride nanotubes (PEG-g-BNNTs), has enhanced dispersion within the polymer matrix, leading to substantial improvements in modulus and tensile strength. Similarly, the use of functionalized carbon-based nanostructures, including GO@HEMA and PEG-GO, improved mechanical properties and electrical conductivity, key parameters that correspond with the electrophysiological nature of bone tissue. However, beside the numerous advantages brought by nanocomposite formulations there are still shortcomings associated with filler agglomeration that can negatively affect the mechanical integrity and homogeneity of the scaffolds.

The integration of artificial intelligence (AI) in tissue engineering (TE) represents a revolutionary step forward in addressing challenges in scaffold design, manufacturing, and performance evaluation. With the exponential growth of biological data and the increasing complexity of engineered tissues, traditional trial-and-error methods are becoming insufficient. AI, particularly through machine learning (ML) and deep learning (DL) approaches, offers a data-driven, systematic alternative capable of accelerating innovation, reducing costs, and improving precision throughout the TE workflow.

One of the most compelling applications of AI resides in scaffold optimization. Utilizing tools such as the finite element method (FEM), AI algorithms can simulate the mechanical behavior of scaffolds based on their microarchitecture and material properties before physical prototyping. This predictive modeling dramatically reduces time and resource consumption while enabling the customization of scaffolds for patient-specific anatomical and functional requirements. Moreover, as demonstrated by studies incorporating ML in design loops like the design-build-test-learn (DBTL) model, researchers can now constantly refine biomaterials using feedback from high-throughput experimental data and algorithmic learning, significantly enhancing scaffold performance. Additionally, AI significantly contributes and can boost characterization and biological performance prediction. ML tools can process vast datasets from imaging techniques (CT, MRI, and SEM) and spectroscopic analyses to extract substantial patterns regarding scaffold–cell interactions, degradation profiles, or biomechanical behavior. This is particularly relevant for PPF-based scaffolds, which present complex correlations between fabrication parameters, mechanical integrity, and biodegradability. AI models tailored for PPF systems could facilitate scaffold tailoring for specific tissues.

In summary, this chapter demonstrates that PPF enables the development of scaffolds that meet both structural and biological demands. Our findings support the transition from stochastic to engineered scaffold architectures, contributing to the field’s movement toward more predictive, tunable, and patient-specific regenerative therapies. By adopting AI-assisted workflows, researchers can not only accelerate innovation but also create smarter, more responsive scaffolds tailored for clinical success. Particularly for PPF-based systems, AI may prove to be the catalyst that enables their full potential in regenerative medicine.

## 6. Conclusions

- Advantages such as biocompatibility and biodegradability to nontoxic products following non-enzymatic hydrolysis of the crosslinking polymer makes PPF a good candidate for medical field, especially for bone tissue engineering.
- Traditional methods like porogen leaching and gas foaming have limitations in controlling essential scaffold parameters, while advanced processing methods such as stereolithography (SLA), microstereolithography (µSLA), and digital light processing (DLP) offer higher resolution and control over scaffold architecture.
- Additive manufacturing techniques enable the fabrication of scaffolds with complex architectures and tailored mechanical properties.
- Post-processing conditions are crucial for maintaining mechanical strength, as demonstrated by the significant drop in properties without adequate post-processing.
- Despite the numerous advantages provided by the 3D printing techniques, there are still deficiencies and drawbacks that need to be considered, mostly coming from the high viscosity of PPF and weak inherent mechanical properties, which limit its processability and use in load-bearing applications.
- Blending PPF with other synthetic or natural polymers or reinforcing it with inorganic and organic substances represents a valuable solution to developing advanced composite formulations to overcome the main limitations.
- Amongst the inorganic reinforcing agents, calcium phosphate enhances bone regeneration and maintains scaffold porosity; hydroxyapatite significantly improves compressive strength and osteoconductivity, leading to well-defined layers and linked pores in 3D-printed scaffolds.
- Bioactive glass composites show good bioactivity and stable mechanical properties over a 12-week degradation period, while boron nitride nanotubes (BNNTs) substantially enhance the mechanical properties of PPF, outperforming other reinforcing agents, such as single-walled carbon nanotubes (SWCNTs).
- In the case of carbonaceous nanostructure PPF-based composites, functionalization proved to be an effective approach to reach optimum mechanical and biological activity of the final materials.
- Graphene oxide nanoribbons and nanoplatelets exhibit high reinforcement potential due to their shape and surface area, surpassing traditional carbon nanotubes in some cases. The reinforcement efficiency of nanoparticles alongside PPF depends on their shape, with nanoplatelets generally providing the most significant reinforcement, followed by nanoribbons and nanotubes. However, considering all these aspects, reinforced PPF can achieve mechanical properties comparable to natural bone, maintaining biocompatibility and promoting cell adhesion and growth.
- By focusing on these prospects, bone tissue engineering can fully utilize AI to advance scaffold production and characterization. This novel approach will not only speed up discovery and development but will also make tissue-engineering structures more effective, dependable, and patient-specific, paving the way for game-changing therapeutic applications.

## 7. Future Perspective

The requirements of a tissue for regeneration in terms of biodegradability, mechanical strength, functionalization, and 3D fabrication should still be the main emphasis of future research in this area of tissue replacement and regeneration. The clinical translation of personalized and patient-specific PPF-based scaffolds should be made easier by the development of innovative fabrication techniques, particularly photo-crosslinked solid-cured 3D printing and hydrogel bioprinting, particularly for defects with intricate geometric and mechanical requirements. Furthermore, the creation of customized individual treatments based on the distinct genetic and molecular composition of every patient holds promise for PPF/PPF composite scaffold fabrication. The design and optimization of nanostructures combined with AI and ML can speed up the development of new medicinal approaches. Moreover, ethical considerations, including data protection and the need for trustworthy data management system, will be critical in shaping future advancements.

## Figures and Tables

**Table 1:** Porosity and pore size values that can be achieved with different synthetic polymers suitable for bone tissue engineering.

Polymer	Porosity	Pore Size	Ref.
PLGA	~83%	180–600 μm	[31]
PCL	70–93%	300–600 μm	[18,32]
PLA	86–90%	N/A	[33]
PPF	~50%	300–900 μm	[34]

**Table 2:** The main techniques used for the design of PPF-based scaffolds.

Material Formulation	Fabrication Technique	Challenges	Key Properties	Ref.
PPF/DEF and BAPO	3D printing-stereolithography	Shrinkage upon polymerization	Compressive modulus ~80–140 MPa Pore size: 446–913 µm Porosity: 35–70%	[42]
PPF and PCL polyphosphoester (PPE)	Solvent casting	Reproducibility and consistency	Biocompatibility, enzyme-responsive degradation, stem cell differentiation	[53]
PPF/DEF-hydroxyapatite (HA) and BAPO	3D printing-micro-stereolithography	Shrinkage upon post curing ~20%	Pore size: 330–360 μm HA facilitate cell adhesion and proliferation	[54]
PPF/DEF	3D printing-micro-stereolithography	Scaffolds shrank 25% after post curing; hydrophobic surfaces	Functionalization with peptides improve cell adhesion and proliferation	[55]
Poly(butyl fumarate)/PPF-DA, nano-HAP composites	Injection molding	Cell viability after 5 days; control over the crosslinking process;	Compressive strengths of 4–12 MPa and compressive modulus of 100–500 MPa; cell viability between 81–90% after one day of incubation	[56]
PPF/DEF and calcium phosphates	3D printing-stereolithography	Calcium phosphate microstructure affects binding capacity for bone-related proteins	Scaffolds coated with calcium phosphates show good osteointegration and osteoconductivity and sustain the release of recombinant human bone morphogenetic protein-2 (rhBMP-2)	[57]
PPF and PPF-co-caprolactone copolymer	Injection molding	Polymerization time of 15–30 min; maximum crosslinking temperature between 38–42 °C	Porosity of 50%; high degradation rate and osteogenesys for copolymer in comparison with PPF	[58]
PPF and hyperbranched polyester acrylate (HPA)	3D printing-projection micro-stereolithography (PμSL)	Post-curing process	Lower shrinkage, higher stiffness and toughness; porosity 80–85%; printing resolution of 50 µm and ultra-fast printing speed of 18 cm/h	[59]
Carboxy terminal-PPF/DEF, BAPO, AND nanohydroxyapatite (nHAP)	Solvent casting	Reproducibility and consistency	In vivo infection model demonstrated that composite scaffolds loaded with antibiotics reduce the bacterial burden at the infection site.	[60]

**Table 3:** The main differences between SLA, DLP, and FDM [73,74,75].

3D Printing Technique	Printing Speed	Printing Resolution XY	Advantages	Limitations
SLA	Moderate (laser traces each point)	~50–140 µm	High surface smoothness (~2 μm roughness); great for complex geometri es; slower than DLP	90% success rate; post-processing operations
DLP	Fast (entire layer cured at once)	~35–100 µm	High resolution and speed; ideal for micro-scale scaffolds, accurate pore geometry; suitable to develop bone tissue substitutes	Limited to photopolymers; post-processing
FDM	Varies, generally slower for detailed prints due to layer-by-layer deposition of material	~100–300 µm	Cost-effective, easy to use; lower resolution, not suitable for intricate bone scaffolds	Prone to failure due to nozzle clogs; 80% success rate

## Data Availability

Data sharing is not applicable to this article as no new data were created or analyzed in this study.
